# Silencing CDK6-AS1 inhibits LPS-induced inflammatory damage in HK-2 cells

**DOI:** 10.1515/med-2021-0314

**Published:** 2021-08-26

**Authors:** Ling Wu, Rui Zhang, Sheng Lin, Min Lin, Jing Wang

**Affiliations:** Department of Pediatrics, Fujian Maternity and Child Health Hospital, Fuzhou 350001, Fujian, China; Pediatric Intensive Care Unit, Fujian Maternity and Child Health Hospital, Fuzhou 350001, Fujian, China; Department of Nosocomial Infection Management, Fujian Maternity and Child Health Hospital, No. 18 Daoshan Road, Gulou District, Fuzhou 350001, Fujian, China

**Keywords:** long non-coding RNA, LPS, CDK6-AS1, apoptosis

## Abstract

In this study, we aim to discover the importance of long non-coding RNA cyclin-dependent kinase 6 (CDK6)-AS1 in lipopolysaccharide (LPS)-induced HK-2 cells. We treated the HK-2 cells with LPS and knocked down CDK6-AS1 in HK-2 cells and then analyzed the effects of CDK6-AS1 on the viability of cell, cell apoptosis, the expression of cytokines via MTT, flow cytometry, enzyme-linked immunosorbent assay (ELISA), and qPCR. The results showed that silencing CDK6-AS1 alleviated LPS-induced inhibition of HK-2 cell proliferation, release of IL-1β, IL-8, IL-6, and TNF-α, cell apoptosis, and decrease in mitochondrial membrane potential. In addition, decreasing the level of CDK6-AS1 inhibited the reduction of Bcl-2 levels, the expression of Bax, cleaved caspade-9, and cleaved caspase-3, induced by LPS. In conclusion, lowering CDK6-AS1 level alleviates LPS-induced inflammatory damage in HK-2 cells.

## 1 Introduction

The rapid development and significant innovations in high-throughput sequencing technology have improved our understanding of the transcriptome. Thirty to fifty percent of the human transcriptome is translated, indicating that non-coding RNAs occupy a very important part of the whole transcriptome. The roles of these non-coding RNAs in human physiological functions are increasingly being explored through research [1]. Compared with the well-understood microRNAs (miRNAs), long non-coding RNA (lncRNA) is attracting increasing attention. lncRNAs are a series of non-coding RNAs over 200 nucleotides in length [2,3]. Recent studies have shown that lncRNAs play important roles in gene transcription, translation, and regulation of other physiological functions [4,5].

Sepsis is a systemic inflammatory response syndrome caused by infection, and is a serious life-threatening complication of disease. When the body reacts to an infection, the reaction can damage the tissues and organs. To date, sepsis has been occurring with increasing frequency, and clinical symptoms have been becoming increasingly serious, resulting in an extremely high current incidence rate and mortality. The current estimated mortality rate of severe sepsis is 28–50% [6,7]. Currently, most research on sepsis-related gene expression continue to focus on the functions of genes that encode proteins. Little is known about the roles of non-protein coding RNAs. Previous studies have confirmed miR-16, miR-223, miR-483-5p, and miR-146a as potential biomarkers for the diagnosis and treatment of sepsis [8,9]. Previous studies have also found that lncRNA HOTAIR, NEAT1, and MALAT1 were differentially expressed in sepsis patients [10,11,12,13]. Involvement of lncRNA cyclin-dependent kinase 6 (CDK6)-AS1 in the pathogenesis and symptoms of sepsis has not been reported. Furthermore, kidney injury and dysfunction are common complications in patients with sepsis [14], and lipopolysaccharide (LPS) induces cell damage and promotes the release of inflammatory factors in kidney [15,16]. Therefore, in this study, we investigated the effects of lncRNA CDK6-AS1 on LPS-induced inflammation in HK-2 cells.

## 2 Methods

### 2.1 Cell culture

HK-2 cells (Xiamen Immocell Biotechnology Co., Ltd, Xiamen, China) were resuspended in the DMEM and cultured in a 5% CO_2_ incubator at 37°C. When the cells were approximately 80% confluent, they were digested with 0.1% trypsin and subcultured.

### 2.2 Construction of plasmid

pLVshRNA-Puro vector (Xiamen Anti-hela Biological Technology Trade Co., Ltd, Xiamen, China) was used to construct a plasmid for knockdown of CDK6-AS1, named shCDK6-AS1. pLVshRNA-Puro vector was used as negative control (shNC). The primers are listed in Table 1.

**Table 1:** The primers for construction of plasmid

Name	Sequence (5′–3′)
shCDK6-AS1-F	CCGGGGAGCAGCACTGCAAGCTATTCTCGAGAATAGCTTGCAGTGCTGCTCCTTTTT
shCDK6-AS1-R	AATTAAAAAGGAGCAGCACTGCAAGCTATTCTCGAGAATAGCTTGCAGTGCTGCTCC

F: forward primer, R: reverse primer.

### 2.3 Blood sample collection

This study was approved by the Ethics Committee of Fujian Provincial Maternity and Child Healthcare Hospital and conducted in accordance with the Helsinki Declaration. All volunteers signed the informed consent forms. Peripheral venous blood (5 mL) was obtained from all the volunteers under fasting conditions in the morning using vacuum vascular collection.

### 2.4 RNA extraction and quantitative PCR (qPCR)

RNA was extracted by using an RNA extraction kit (Epoch Life Science Inc., catalog number: 1660050). After total RNA was reverse transcribed into cDNA with reverse transcription kit (Roche, catalog number: 11939823001), qPCR was performed using SYBR Green Master Mix (Vazyme, catalog number: Q111-02). The reaction conditions of qPCR are as follows: 95°C for 30 s followed by 40 cycles of 95°C for 5 s, 60°C for 34 s, 70°C for 10 s, followed by 95°C for 15 s. The primers are shown in Table 2. The data are expressed as the mean value ± standard deviation (SD) of three independent experiments.

**Table 2:** The primers for qPCR

Name	Sequence (5′–3′)
CDK6-AS1-QF	GCTGATGATGCCTCTTGT
CDK6-AS1-QR	TTAATCTCAAAGTGGCTGA
18S rRNA-QF	ACCCGTTGAACCCCATTCGTGA
18S rRNA-QR	GCCTCACTAAACCATCCAATCGG
IL-1β-QF	CCACAGACCTTCCAGGAGAATG
IL-1β-QR	GTGCAGTTCAGTGATCGTACAGG
IL-6-QF	AGACAGCCACTCACCTCTTCAG
IL-6-QR	TTCTGCCAGTGCCTCTTTGCTG
IL-8-QF	GAGAGTGATTGAGAGTGGACCAC
IL-8-QR	CACAACCCTCTGCACCCAGTTT
TNF-α-QF	CTCTTCTGCCTGCTGCACTTTG
TNF-α-QR	ATGGGCTACAGGCTTGTCACTC
AL022100-QF	GGACAAAGCCATCGGAGAA
AL022100-QR	TTGGTCAGAGCCCAGCAT
AL353747-QF	GCGATGGCTCCACTGACT
AL353747-QR	TGAGGTGCTCGTGTTGCT
AC005871-QF	CCTCATCACGGACCTATC
AC005871-QR	AAGTGCGTTGTCATTACCT
AC008494-QF	AACAGAAACCCGAGAATA
AC008494-QR	AGTAAAGGAAAGGCAAAG
AC084125-QF	ATGCCTCCGTCACGCCTCT
AC084125-QR	TCTGTACTTCCCATCCTGTCC
AC087477-QF	GCCGCCAGGACTTCACTT
AC087477-QR	ATGCTTCTGCTCCCAAAT
AC002558-QF	ATGTCCCAACAATGAAAG
AC002558-QR	AATCTTATCTGGTGGAGTG
AC009145-QF	GAAAGGACCTCATGCAAAG
AC009145-QR	GATGGGTAAACAGAATCAAGC
AC021915-QF	GACAGTAGCACCCACCTC
AC021915-QR	CACAGCATCCTGAACCCT
AC025160-QF	AAGCCATATCTTCTACAACTC
AC025160-QR	AACTTTCCCTGTCACCTAC
AC099328-QF	TGGGAACTTGATACCTGA
AC099328-QR	GAGGCTTCATCGAAAGAG
AP001007-QF	CAGCCCATCTCCGCTCCACT
AP001007-QR	TCTCCGCAGCCTCGTCTT
MIR100HG-QF	CATAAACTTGGCTTCCTC
MIR100HG-CQR	AAACCTGCTTCCATCTTG
TM4SF19-AS1-QF	CCTCCACCCATTTACCTAC
TM4SF19-AS1-QR	AGCCCTGATTTGCTTTGT

QF: forward primer for qPCR, QR: reverse primer for qPCR.

### 2.5 MTT assay

Cells were plated in 96-well plate with 100 μL per well. After adhering to the wall, the cells were incubated for 0, 12, 24, or 48 h with LPS of 0, 1, 5, or 10 μg/mL, or treated with 1% DMSO (DMSO group), 5 μg/mL LPS (LPS group), 5 μg/mL LPS plus shNC (LPS + shNC group), or 5 μg/mL LPS plus shCDK6-AS1 (LPS + shCDK6-AS1) for 24 h. Subsequently, 20 μL MTT solution (5 mg/mL) was added to each well. After 4 h of culture at 37°C, 150 μL of DMSO was added to each well. Plates were agitated at low speed on a shaking table for 10 min to fully dissolve the purple crystals. Absorbance was detected at 570 nm using a microplate reader. The data are represented in terms of mean value ± SD for sextuple wells.

### 2.6 Flow cytometry analysis

Cells were cultured overnight after plating at a concentration of 3 × 10^5^ cells/well. Then, the cells were treated with 1% DMSO, 5 μg/mL LPS, 5 μg/mL LPS plus shNC, or 5 μg/mL LPS plus shCDK6-AS1 for 24 h. Cells were collected by centrifugation and washed twice with pre-chilled phosphate buffer saline. The cells were stained using the JC-1 staining assay kit (Beyotime, catalog number: C2006) or the annexin V-FITC/propidium iodide (PI) apoptosis detection kit (Vazyme, catalog number: A211-01) according to the manufacturer’s instructions. Subsequently, mitochondrial membrane potential and apoptosis were detected via flow cytometry. Three independent experiments were performed.

### 2.7 Western blotting

After total proteins from the cells were extracted with RIPA buffer (Beyotime, Catalog number: P0013C), quantified with the BCA protein concentration determination kit (Beyotime, catalog number: P0012S), and separated by SDS-PAGE, western blotting was performed, as previously described in ref. [17]. The antibodies used for western blotting are listed in Table 3. The experiments were performed thrice independently.

**Table 3:** Antibodies for western blotting

Classification	Name of antibody	Manufacturer	Catalog number	Dilution rate
Primary antibody	Bcl-2 antibody	Cell signaling technology	15071	1:1,000
	Bax antibody		89477	1:1,000
	GAPDH antibody		97166	1:1,000
	Cleaved caspase-3 antibody		9664	1:1,000
	Cleaved caspase-9 antibody		20750	1:1,000
Secondary antibody	HRP-conjugated affinipure anti-mouse IgG		7076	1:1,000
	HRP-conjugated affinipure anti-rabbit IgG		7074	1:1,000

### 2.8 Enzyme-linked immunosorbent assay (ELISA)

After the cells were treated with 1% DMSO, 5 μg/mL LPS, 5 μg/mL LPS plus shNC, or 5 μg/mL LPS plus shCDK6-AS1 for 24 h, the supernatant was collected to analyze the IL-1β, IL-6, IL-8, and TNF-α levels using the corresponding ELISA kit according to the manufacturer’s instructions. The details of ELISA kits are shown in Table 4. The experiments were performed thrice independently.

**Table 4:** The information of ELISA kits

Name	Catalog number	Manufacturer
Human IL-1β ELISA Kit	PI305	Beyotime
Human IL-6 ELISA Kit	PI330	
Human IL-8 ELISA Kit	PI640	
Human TNF-α ELISA Kit	PT518	

### 2.9 Statistical analyses

Experimental data were analyzed using origin 8.5. Student’s *t* tests were used for two group comparisons. One-way ANOVA was used for comparison between multiple groups. Cell counting data were expressed as percentages, and the chi-square test was used for comparison between groups. *P* < 0.05 was considered statistically significant.

## 3 Results

### 3.1 LPS induces the increase in CDK6-AS1 level in HK-2 cells

**Figure 1:**
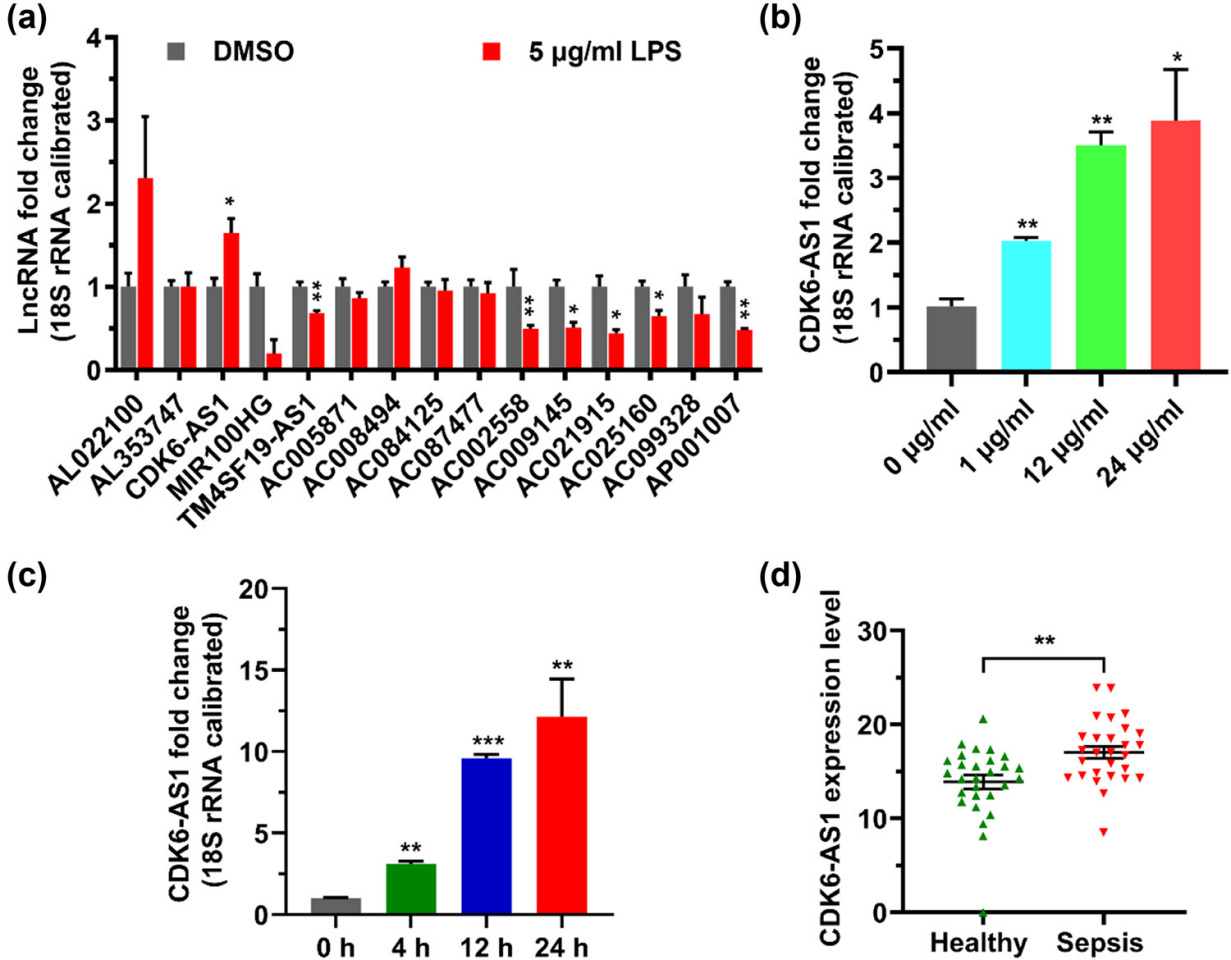
Effect of LPS on CDK6-AS1 expression: (a) effect of LPS on lncRNA expression, (b) CDK6-AS1 level in cells exposed to different LPS concentrations, (c) CDK6-AS1 level in cells after LPS treatment for various times, and (d) expression levels of CDK6-AS1 in blood. *P < 0.05, **P < 0.01, ***P < 0.001.

To investigate the effect of LPS on the level of lncRNA in cells, HK-2 cells were stimulated by LPS and the changes in 15 lncRNA levels in cells were detected by qPCR. Compared with negative control, CDK6-AS1 level in cells treated with LPS was significantly increased (Figure 1a). Moreover, the results showed that LPS upregulated CDK6-AS1 in a dose-dependent and time-dependent manner (Figure 1b and c). In addition, CDK6-AS1 levels in the blood of sepsis patients were higher than those in healthy donors (Figure 1d). Therefore, this study chose to verify the effect of CDK6-AS1 on LPS-induced inflammation in HK-2 cells.

### 3.2 Decreasing CDK6-AS1 inhibits the destruction of cell viability by LPS

**Figure 2:**
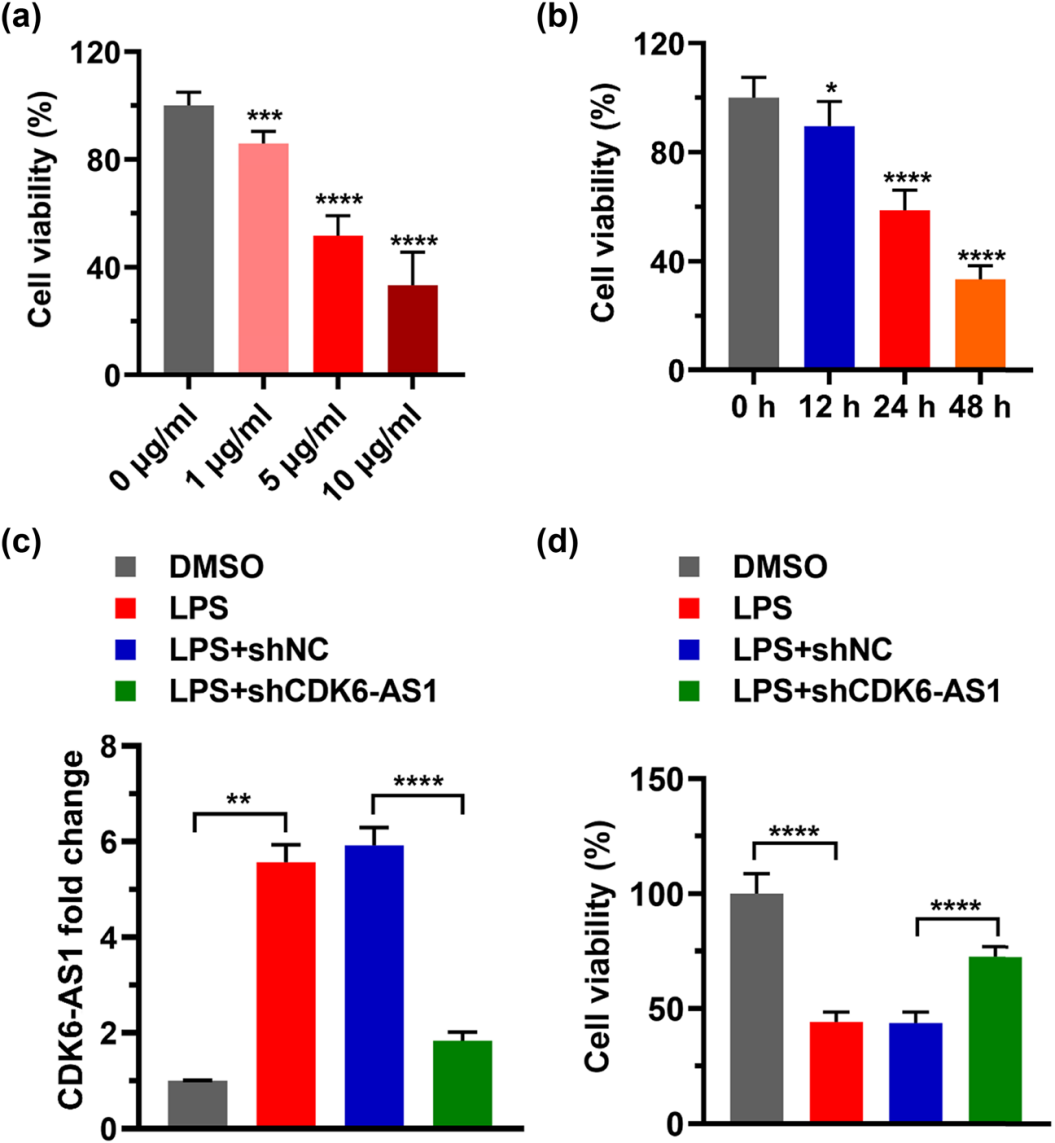
Effects of LPS and CDK6-AS1 on cell viability: (a) cell viability after treatment with various concentrations of LPS, (b) cell viability after LPS treatment for various times, (c) relative levels of CDK6-AS1 expression, and (d) effect of LPS plus CDK6-AS1 silencing on cell viability. **P < 0.01, ***P < 0.001, ****P < 0.0001.

To study the role of LPS and CDK6-AS1 in cell viability, we stimulated cells with reduced CDK6-AS1 levels using LPS and then detected cell viability using MTT assay. The viabilities of cells treated with 1, 5, and 10 μg/mL of LPS were significantly lower than that of the control (Figure 2a). Based on dose ranging results, we selected 5 μg/mL of LPS for subsequent experiments. HK-2 cells were treated with 5 μg/mL of LPS for 0, 12, 24, and 48 h. The MTT assay showed that cell viability decreased significantly with the increase in treatment time (Figure 2b). Based on these results, we chose 24 h of LPS treatment for subsequent experiments. Next CDK6-AS1 level was knocked down in HK-2 cells. Compared with the DMSO group, the level of CDK6-AS1 in the LPS group was significantly higher (Figure 2c). Compared with the LPS + shNC group, the CDK6-AS1 level in the LPS + shCDK6-AS1 group was significantly lower (Figure 2c). These results indicated that LPS positively regulated CDK6-AS1. The cell viability in LPS group was significantly lower than that in DMSO group, and the cell viability in LPS + shCDK6-AS1 group was significantly higher than that in LPS + shNC group (Figure 2d). These data suggested that LPS inhibited cell viability by targeting CDK6-AS1. Together, these findings suggested that silencing CDK6-AS1 suppresses the destruction of cell viability by LPS.

### 3.3 Lowering the level of CDK6-AS1 alleviates LPS-induced apoptosis

**Figure 3:**
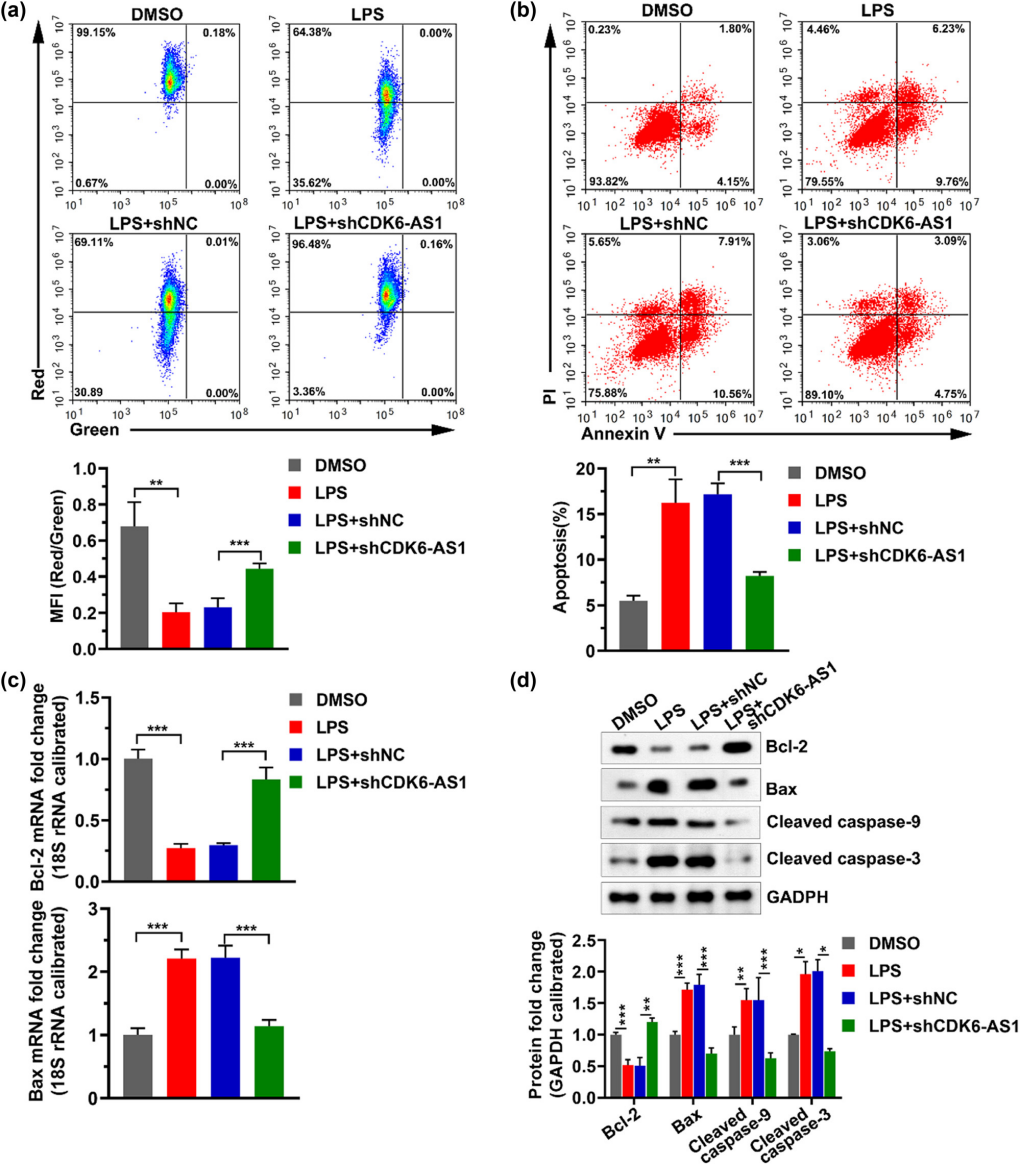
Effects of LPS and CDK6-AS1 on apoptosis: (a) the representative image of mitochondrial membrane potential and the histogram of statistical mean fluorescence intensity (MFI), (b) the representative image of apoptosis and the histogram of apoptosis rate, (c) the mRNA levels of Bcl-2 and Bax detected by qPCR, and (d) protein levels of Bcl-2, Bax, cleaved caspase-9, and cleaved caspase-3 tested by western blotting. MFI: mean fluorescence intensity. PI: propidium iodide. *P < 0.05, **P < 0.01, ***P < 0.001.

Previous studies have shown that LPS induces inflammation and apoptosis by downregulating Bcl-2 expression and increasing the levels of Bax and Caspase-3 [18]. To explore the role of CDK6-AS1 and LPS in apoptosis of HK-2 cells, we used JC-1 staining and the annexin V-FITC/PI staining to detect the mitochondrial membrane potential and cell apoptosis, respectively, and used qPCR and western blotting assays to teste the levels of apoptosis-related marker genes. The result showed that LPS resulted in a decrease in mitochondrial membrane potential, while downregulating CDK6-AS1 level in cells relieved the decrease of mitochondrial membrane potential caused by LPS (Figure 3a). Flow cytometry results showed that the number of apoptotic cells in HK-2 cells treated with LPS was significantly higher than that in cells not treated with LPS, while silenced CDK6-AS1 significantly inhibited LPS-induced apoptotic (Figure 3b). LPS significantly reduced the mRNA and protein levels of Bcl-2, and significantly increased the mRNA and protein levels of Bax, while silencing of CDK6-AS1 mitigated the effects of LPS on the mRNA and protein levels of Bcl-2 and Bax (Figure 3c and d). In addition, LPS significantly increased the levels of cleaved caspase-3 and cleaved caspase-9, while decreasing CDK6-AS1 inhibited this effect of LPS (Figure 3d). These data revealed that downregulating CDK6-AS1 level alleviated LPS-induced apoptosis.

### 3.4 Downregulating CDK6-AS1 level alleviates LPS-induced inflammatory cytokine

**Figure 4:**
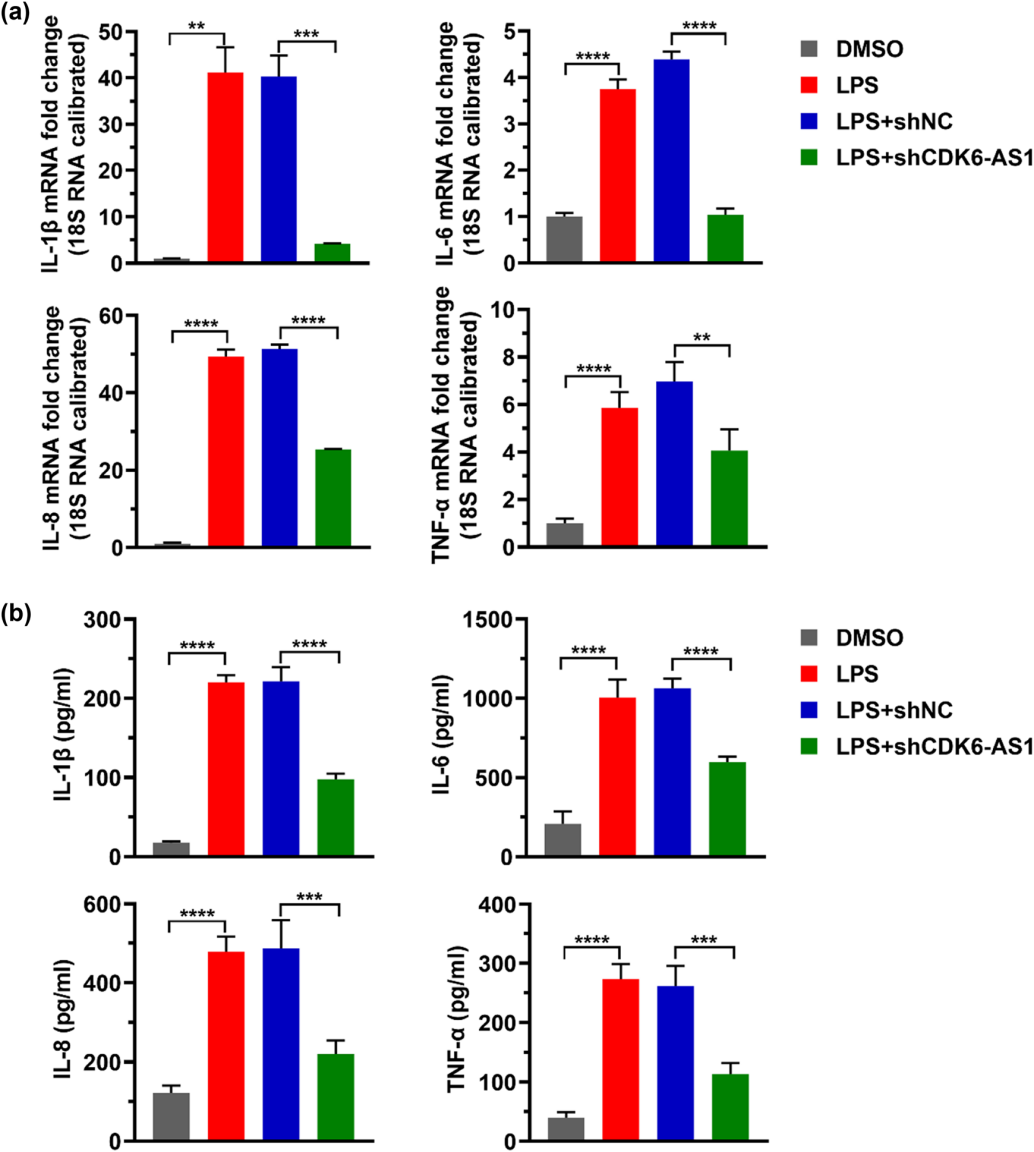
Effects of LPS and CDK6-AS1 on inflammatory cytokine: (a) the mRNA levels of IL-1β, IL-6, IL-8, and TNF-α detected by qPCR and (b) the secretion levels of IL-1β, IL-6, IL-8, and TNF-α detected by ELISA assay. **P < 0.01, ***P < 0.001, ****P < 0.0001.

LPS has been reported to cause an inflammatory response and induce the production of inflammatory cytokines [19]. In order to investigate the effect of CDK6-AS1 on LPS-induced inflammatory factors, we detected the levels of IL-1β, IL-6, IL-8, and TNF-α using qPCR and ELISA. The result indicated that LPS enhanced the mRNA level and secretion level of IL-1β, IL-6, IL-8, and TNF-α, while lowering the CDK6-AS1 level attenuated the LPS-induced production of IL-1β, IL-6, IL-8, and TNF-α (Figure 4).

## 4 Discussion

At present, studies of lncRNA expression in sepsis mainly focus on HOTAIR, MALAT1, NEAT1, TUG1, and UCA1 [20]. Previous studies have shown that HOTAIR is upregulated in sepsis models [21]. The expression level of MALAT1 was significantly increased in sepsis *in vitro*, and MALAT1 knockout also reduced serum levels of cTn-I, TNF-α, IL-1β, IL-6, IL-10, IL-17, IFN-γ, C5, and C5a [10]. NEAT1 expression was significantly increased in patients with sepsis-induced acute kidney injury and correlated positively with severity of sepsis [22]. However, the role of CDK6-AS1 in sepsis-induced kidney injury is rarely reported. In this study, we demonstrated that silencing CDK6-AS1 inhibited LPS-induced inflammatory response in renal epithelial cells *in vitro*, laying the foundation for further investigation on the role of CDK6-AS1 in sepsis-induced renal injury.

Previous studies have shown that CDK6 is a regulator key of cell cycle [23]. However, in current study, we showed that apoptosis was significantly increased by LPS in HK-2 cells normally expressing CDK6-AS1, and silencing of CDK6-AS1 significantly attenuated this effect of LPS. These results showed that CDK6 and CDK6-AS1 play different roles. Apoptosis is considered to be a key part of septic-induced acute kidney injury [16]. Many molecules are involved in apoptosis, including Bcl-2, Bax, caspase-9, and caspase-3 [24]. Our study showed that LPS inhibits the expression of Bcl-2, and promotes the expression of Bax and the cleavage of caspase-9 and caspase-3 via upregulating the level of CDK6-AS1.

LPS stimulation leads to severe inflammation, which produces a large number of inflammatory factors, such as TNF-α, and further induces apoptosis and kidney damage [16]. Our results demonstrated that LPS promoted the expression of IL-1β, IL-6, IL-8, and TNF-α by regulating CDK6-AS1.

Due to the lack of animal models of acute kidney injury induced by sepsis in this study, it is not impossible to further reveal the role of CDK6-AS1 in acute kidney injury induced by sepsis, which is the limitation of this study.

In conclusion, in HK-2 cells, LPS induced an increase in CDK6-AS1 levels, while silencing CDK6-AS1 inhibited the secretion of inflammatory factors and inhibited cell apoptosis induced by LPS, indicating that silencing CDK6-AS1 in HK-2 cells alleviated the LPS-induced inflammatory damage.
